# Relevance of the Health Extension Program to the current Health Needs and Evolving Demands of Rural Ethiopia: A Mixed-Method Analysis

**DOI:** 10.4314/ejhs.v33i1.8S

**Published:** 2023-04

**Authors:** Wasihun Andualem Gobezie, Girmay Medhin, Mulusew G Jebena, Mekdes Demissie, Yibeltal Kiflie Alemayehu, Alula M Teklu, Girma Azene, Tegene Legese Dadi, Million Tesfaye

**Affiliations:** 1 MERQ Consultancy PLC, Addis Ababa, Ethiopia; 2 Averting Maternal Death and Disability (AMDD), Columbia University; 3 Addis Ababa University, Aklilu Lemma Institute of Pathobiology; 4 Jimma University, Department of Epidemilogy, Jimma; 5 Centre for Innovative Drug Development and Therapeutic Studies for Africa (CDT-Africa), College of Health Science, Addis Ababa University; 6 College of Health and Medical Sciences, Haramaya University, Ethiopia; 7 Department of Health Policy and Management, Jimma University, Ethiopia; 8 Department of Global Community Health and Behavioral Sciences, School of Public Health and Tropical Medicine, Tulane University, New Orleans, USA; 9 Hawassa University, College of Medicine and Health Science, School of Public Health

**Keywords:** Relevance, evolving demands, health extension program, health needs, rural Ethiopians

## Abstract

**Background:**

Ethiopia has been implementing a health extension program (HEP) to respond to the high maternal and child mortality in rural communities. HEP has brought tremendous contributions to improved access and coverage of primary healthcare in the last 15 years. Despite its attributions, attention to HEP has declined in recent years due to several reasons. This study is designed to explore HEP's relevance to the current healthcare needs of the rural communities.

**Methods:**

This study is a nested cross-sectional mixed-method to the overall HEP's evaluation between March and May 2019. Descriptive statistics were used on qualitative and quantitative assessment. A literature review supplemented the assessment. A representative quantitative sample of 11,746 women, men, and young girls; a qualitative sample of 268 key informants from Kebele administrators, HEWs, program people in the health system and focus groups from community leaders, men and women from 185 Kebeles in 62 woredas were selected. A thematic approach was used for qualitative analysis.

**Results:**

Rural communities and program managers asserted that HEP's service packages with the existing service delivery modalities were relevant. Eighty-two percent of men and women and 77% of young girls confirmed this relevance. Besides the existing packages, additional curative services for adults and children were recommended with emphasis on the pastoralist community. HEP's service uptake has declined as over 86% of targeted rural communities bypassed HPs due to unavailability of services and capacity problems of HEWs.

**Conclusion:**

The current HEP packages with the existing service delivery modalities are still relevant to the rural communities' health needs. However, on-going changes to address the evolving demands of the targeted rural communities are crucial.

## Introduction

In the 1990s, Ethiopia suffered from famine coupled with decades of civil war and was burdened with an inefficient health system incapable of addressing the priority health needs of rural communities (1, 2). Most of the programs established between 1998 and 2002 were poorly integrated, under-funded, and had a design that could not address access and coverage of the rural population which made up 85% of the population (1). There were unacceptably high maternal, neonatal, and child mortality due to causes that are highly preventable (3,4).

In 2017, The Global Burden of Disease study showed that communicable diseases, maternal, neonatal, and nutritional disorders (CMNNDs), non-communicable diseases (NCDs), and injuries accounted for 60%, 33%, and 7% of the country's total disability adjusted life years (DALYs), respectively (20), indicating that the country is affected by such disease burdens.

To address such high morbidities and to work towards universal primary healthcare coverage (5), the Ethiopian government established the health extension program (HEP). The HEP was initiated in agrarian areas in 2003 with 16 health service packages under five categories (1, 2). The five categories of HEP were personal hygiene and environmental sanitation, major communicable and neglected tropical diseases (NTDs), family health services, non-communicable diseases (NCDs), and health education and communication. The packages were considered relevant by the policy makers to the rural communities. The packages have been delivered through outreach, home visits, and static approaches (2, 6, 7).

The HEP was initially piloted in a few *Woredas*/districts of Tigray (8) and then scaled up to the larger regions, Oromia, Amhara, Southern Nations and Nationalities (SNNP), and the remaining districts of Tigray. The program started with pairs of trained female health extension workers (HEWs) in agrarian areas and then expanded to include male HEWs in pastoralists areas in 2006 (9). In 2009, HEP expanded to urban centers with additional curative services (2, 7, 9-11). In 2016, the government added two new service packages that made up a total of 18 packages with additional standards of commodities and training of HEWs (12).

Reflecting its objective of achieving primary healthcare coverage and meeting the Millennium Development Goals (MDGs) (10, 11, 13, 14), HEP has made significant contributions in improving access and coverage of key primary healthcare services in the last 15 years (15-17). Despite that HEP has brought remarkable changes in disease prevention and control, it has suffered from lack of attention from its stakeholders in recent years (18). The political turmoil, changes in government structures, and lack of adaptable leadership plus limited capacity to cope with the evolving health needs attributed to lack of attention (7, 18). Little is known about HEP's relevance. This study is, therefore, designed to answer the question, “Is HEP still relevant to the health needs of rural communities?” The findings of this study will inform policy makers, implementers, funding agencies and the rural community about the relevance of the HEP and its responsiveness to the current health needs.

## Methods

**Study setting**: Ethiopia, with a population of 112 million, is largely agrarian (85%). During the data collection time, the country was divided into 9 regional states and two city administrations. The regions are Afar, Amhara, Benishangul-Gumuz, Gambella, Harari, Oromia, SNNP, Somali and Tigray and the two city administrations are Addis Ababa and Dire Dawa (19).

HEP is implemented in three delivery points: at HPs, through home visits by HEWs, and outreach service deliveries using HEWs and women development army (WDA) leaders (2). One HP is designed to serve a Kebele (lowest administrative unit that accommodates a population size of 3,500 to 5,000) (2).

**Study design**: This study adapted a framework developed by Abdelmagid and colleagues for appropriateness or relevance of humanitarian programs (21). Appropriateness and relevance are interchangeably used in many areas (21). The Organization for Economic Co-operation and Development (OECD) defines “relevance” as “*the extent to which a program's objectives are in line with the community's health needs and priorities*” (22). In the framework, program relevance or appropriateness is measured using three major questions: a) The Who, b) The What, and c) The How (21). This study applied this framework to measure the relevance of the HEP program in relation to these three questions.

### Data Sources, Sampling Procedures, and Sample Size

**Data source**: The study is a cross-sectional, nested within the national assessment of HEP study, conducted from February to May 2019 (23, 33). Multiple data sources were used to develop this paper; a) quantitative data from the HEP assessment, b) qualitative data (in-depth interviews and focus group discussions conducted from community to federal levels), c) data from other secondary sources-disease burden studies, and d) findings from literature reviews.

**Sampling of respondents**: Woredas and kebeles were selected randomly with a design effect imposed to adjust for the smaller and larger regions. A three-stage random sampling was employed to select Woredas, HPs, and members of the targeted rural communities. A random sample of 38 households from each Kebele (185 kebeles) were selected from 62 woredas. Household members (men, women, and young girls) were part of the quantitative survey. Eligibility of the study was generally dependent on availability of a woman greater than 15 years of age (head or wife) in study household and checked for availability of a man with age 15 or more (head or husband) and female youth (age 15 – 24) to be interviewed. If two or more female youth were available in a household at the time of the survey, one of them was randomly selected for interview. Kebele leaders, government and partner organizations, HEWs, service providers in the health centers (HCs), and targeted rural community members were part of the qualitative survey.

**Sample size**: For the quantitative part, a total of 7,043 women, 4,805 men, and 1,020 young girls and WDAs were included in the analysis. WDAs were volunteers that received training on HEP to assist the paid HEWs (23). For the qualitative data, 172 interviews and 109 focus group discussions were conducted; these data were included in the analysis. Focus groups were organized in each of the study Kebele. HEWs and WDAs facilitated the qualitative data collection and selection of the community participants from rosters of HEWs and from Kebele Administration records. A total of 6-12 members were participated in each focus group discussion.

**Systematic review**: For the systematic review of the literature, a search from the AJOL, PubMed, Google Scholar, EMBASE, Ovid, and Scopus databases was conducted by combining “health extension”, “community health worker”, “HP” or “primary healthcare” and “Ethiopia” as search phrases. Gray literature was searched from all academic institutions of the country, with a focus on the HEP and PHC.

**Data collection tools and measurements**: MERQ Consultancy PLC trained and deployed data collectors. The data collection was done with tablets using Open Data Kit version 1.4.1. The data collection tools were adapted from national and international standard survey instruments, such as the Ethiopian demographic and health survey and the Ethiopian service availability and readiness assessment as the questions in these tools were tested over several years. The relevance of HEP was measured based on the parameters outlined in the table below:

**Table 1:** Measurement of relevance of HEP characteristics/parameters, HEP assessment, 2019

Components of the study framework	Parameters/Characteristics	Measurement strategies	Source of data
**The Who - Who is targeted?**			

**Do target communities clearly identified and prioritized based on their health needs?**	HEP targeted rural community in the country	Description of profile of HEP-targeted rural communities in Ethiopia; disaggregated by women, men, and young girls and agrarian and pastoralist setting	Literature review Quantitative and qualitative data

**The What - What service packages?**			

**Are HEP packages relevant to the health needs in 2017?**	Disease burden	Analysis is done on what causes the most death and disability comparing two-time periods (2007 and 2017) that are close to the beginning of the HEP and during the HEP assessment. These times were selected due to availability of data.	Global Disease Burden data for Ethiopia
**Are the HEP packages still relevant to the evolving demands of the rural communities?**	Linkage of HEP packages and current disease burden	To check whether the packages are aligned with the disease burden or not	Literature

**The How - What service delivery modalities?**			

**Are the HEP service delivery modalities acceptable and appropriate to provide HEP packages?**	Home visit by HEWs as a delivery modality	Show whether home visit as a delivery modality is acceptable or not by the rural communities	Quantitative and qualitative data
	Outreach service delivery modality through HEWs/WDAs	Show whether outreach is acceptable or not as delivery modality of HEP	Quantitative and qualitative data
	Health post as a delivery modality	Show whether HP is an acceptable delivery modality of HEP or not	Quantitative and qualitative data

**Data analysis**: Frequencies and cross-tabulations were used to summarize quantitative data. We used 28 sets of questions having responses on the Likert scale (24) to measure the perception of the targeted rural community members towards relevance of service packages and modalities. We used STATA version 14 for data analysis.

The interviews were transcribed and translated into English. A group of 15 social researchers that are experienced with qualitative data analysis in many large-scale surveys were recruited for the analysis. The qualitative researchers developed a codebook based on the preliminary readings of some transcripts. The transcripts were coded using NVIVO 12. Thereafter, a thematic analysis was used to categorize the codes and develop themes (25). Both the quantitative and qualitative data were used to triangulate the findings of this study. The findings of the quantitative data converge with the results of the qualitative in many instances.

**Ethical considerations**: This study, as part of the 2019 HEP assessment, was endorsed by MoH and ethically approved by the Institutional Review Board of the Ethiopian Public Health Institute.

## Results

The results section is organized based on the research questions. We first present the relevance of HEP packages, followed by the health needs of targeted rural communities (men, women, young girls), and lastly how the service delivery modalities are relevant to deliver HEP packages.

### Relevance Of Current Hep Service Packages (The What)

**Community's perception towards the relevance of HEP packages**: From the qualitative findings, program experts and HEWs confirmed that the HEP packages were relevant to the community's health needs. Rural community members were aware of personal hygiene and environmental sanitation due to HEP. The communities used latrines. Reflections from HEWs accentuated the acceptance of the packages by the communities:

*“Previously (before HEP), open field defecation was common in our community; but after the HEP started, many households have constructed latrines.” (Malie Woreda (a HEW from SNNP)*

Some other program experts were also mentioning HEP's contribution that cemented relevance of its packages: “*This program (HEP) created linkages and equity of services. For example, before HEP, communities were traveling long distance to get family planning services in cities; but the services are available now close to our doors. The community's health seeking behavior has been improving over time.” (Program Officer from Oromia RHB)*

Community leaders also agreed that the type and the delivery of HEP packages are relevant to the communities: “*The HEWs have educated us on family planning and hygiene and sanitation. Many women give birth every year (no spacing of birth), and they do not take care of their children. After advised by HEWs, women started using family planning methods, and they are able to space their children.” (A community leader from SNNP)*.

**Figure 2:**
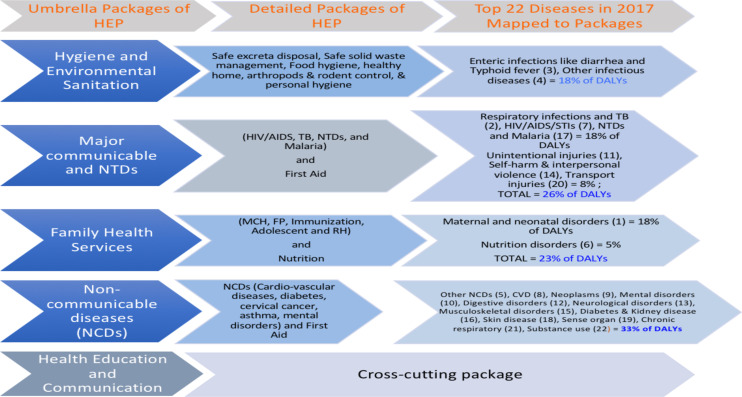
Mapping of current rural HEP service packages to the disease burden, data from literature, GDB 2019 Note: The numbers in the parentheses are ranks in the global burden of disease (GBD) list that cause most death and disabilities

**Mapping of current HEP service packages to Ethiopia's disease burden**: From the recent GBD study in our literature review (Figure 2 above), HEP's packages address non-communicable diseases, which cause 33% of DALYs lost; followed by major communicable diseases and neglected tropical diseases (NTDs) that cause 26% of the total DALYs. Family health service package is also one of the five key HEP packages that contributed to the reduction of 23% of the total DALYs listed in the disease burden for the country. Infectious diseases contribute to 18% of the country's DALYs (20).

### Do all HEP packages reflect the health needs of the target community (for whom)?

**Figure 3:**
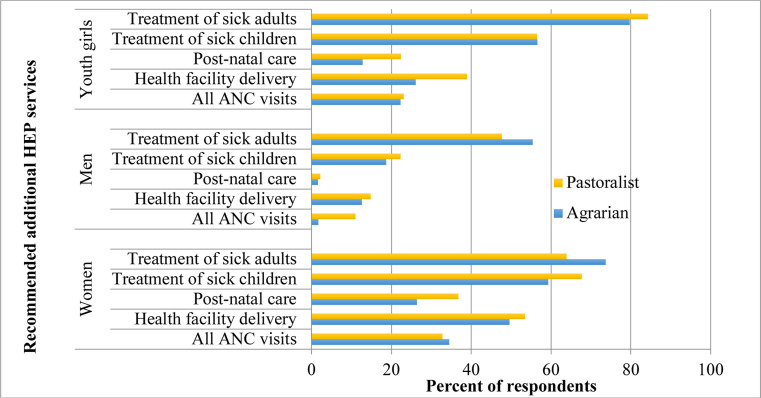
Percentage of household members who have ever visited a HP that recommended additional services in response to their health needs, HEP quantitative data, 2019

The quantitative results show that all the current HEP packages are relevant as the rural communities that were targeted and being served did not propose the dropping of any HEP package (data not shown). Rather, they suggested more services in addition to the existing packages to meet their health needs. This implies that the existing packages were all relevant to them. Figure 3 below shows the additional services they proposed. Treatment of sick adults and sick children were the two most important additions from both agrarian and pastoralist rural communities.

### Relevance of HEP's Current Service Delivery Modalities-The How?

**Relevance of home visits by HEWs**: According to the qualitative findings, rural communities and HEWs mentioned that home visit was relevant. Service delivery through a home visit reduced the occurrence and transmission of disease: “*The HEWs in our Kebele provide vaccination of babies within 45 days traveling home-to-home, which is helpful. For other kids and mothers, HEWs also provide plumpy nut for nutritional support and health education to community members during home-visits (sic) is also appropriate.”* (Mekdela Woreda; a WDA from Amhara)

**Figure 4:**
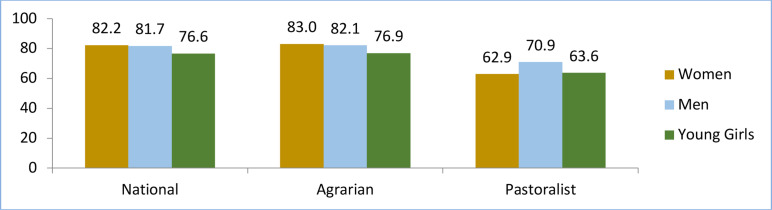
Percentage of household (HH) members who had perceived agreement on the appropriateness of HEWs' service provision at the HH level. (Data from national HEP quantitative survey, 2019)

The quantitative assessment (shown in Figure 4) also converges with the findings of the qualitative assessment. Quite large proportions (82%) of women and men and 77% of young girls agreed that home visit is a relevant service delivery modality.

**Relevance of HP as a service delivery point**: HPs are the closest health facilities to the rural communities. Targeted communities affirmed that HP's were important service delivery points for them. However, they were not receiving the services they sought in the HPs; demanding more curative services than before that resulted in bypassing the HPs to other closest hospitals and private health facilities. The following two quotes reflect the situation:

*“When women and men come to a HP, we diagnose and refer to a HC for further diagnosis and treatment… the (HP) setting is not suitable to provide (sic) some curative services…”* (Mana Buture Woreda; a HEW from Oromia)

*“Currently, if a woman is in labor, a health extension worker calls an ambulance to take her to a health center and deliver there.’* (Raya Kobo Woreda; a woman community member from Amhara)

The quantitative assessment (Table 2) was also in line with the findings of the qualitative findings. Over 86% of targeted rural communities that bypassed the HPs/HEWs cited problems of HP/HEW capacity and service availability.

**Table 2:** Percentages of household members with illness and the treatment sought from health facilities as well as reasons for bypassing HPs/HEWs in the past 12 months by livelihood

	Total number of patients bypassing HPs without referral	Reasons for not having a referral from a HP/HEWs						

		HP was closed or unavailable	HEW was unavailable	Service unavailable	HEWs have no capacity to provide a specific service	No need to go as HEWs refer anyway	HC/Hospital is close by	Other
National Livelihood	822	6.7	7.8	34.0	33.4	4.3	13.6	0.2
Agrarian	660	6.6	7.6	34.0	33.6	4.3	13.7	0.2
Pastoralist	162	10.1	18.0	35.9	22.1	3.1	10.3	0.4

**Relevance of outreach services through HEWs and/or WDA/had leaders**: From the qualitative assessment, HEP service provision through outreach modality is relevant especially for hard-to-reach rural areas. It is also relevant in addressing the large population at a time of campaign activities, such as immunization: “*Vaccination of children is conducted in the form of campaign with support from catchment HC. After such immunization activities, we haven't seen measles cases ever (sic) in our locality*.” Wama Hagalo; a community member from Oromia)

Respondents also gave feedback about the relevance of the outreach service modality for specific community groups that do not commonly access health facilities: “*When HEWs call us for (sic) a meeting and they educate us about negative (sic) impact of unprotected sex. They tell us on how to use condoms. We get tested for HIV every three months, and HEWs visit us regularly…”* (Bullen Woreda; a sex worker from Benishangul-Gumuz)

From the quantitative assessment, nearly one-fifth of women HH members had ever used the outreach service delivery modality for receiving health education or other services at the national level, which supplemented the qualitative result (Table 3).

**Table 3:** Percentage of women HH members who had received health education/other services from an outreach service delivery modality by service type

	Total number of women HH members interviewed	Percentage who ever had visited an outreach service delivery point	Number of women who ever had received services in outreach	Percent received the following services:					

				Health education	Vaccination for children	Growth monitoring	Vaccination for women	Deworming	Supplemental food

	n	%	n	%	%	%	%	%	%
National Livelihood	7,122	21.7	1,419	83.1	39.1	16.8	19.4	18.4	9.8
Agrarian	4,854	22.3	1,175	83.1	39.0	16.7	19.2	18.3	9.8
Pastoralist	2,268	8.0	244	78.4	42.3	18.5	31.0	23.0	15.3

## Discussion

This study provides an insight into the overall picture of HEP's relevance to the current health needs of rural Ethiopians. The use of mixed methods approach helped us to see convergence in the findings. The findings are important as planning, implementation, and monitoring yardsticks to improve strategies in overcoming bottlenecks in the nationwide effort of achieving universal primary healthcare coverage.

The current HEP packages are well mapped to address the priority health needs of the targeted rural communities. Ethiopia is not unique in being hit by multiple disease burdens - it is a common phenomenon in many low and middle-income countries (32). Ethiopia has been continuously adapting the HEP packages by inclusion of some curative services, such as integrated community case management (ICCM), community-based newborn care (CBNC), and treatment of pneumonia, malaria, first aid, chronic illnesses, and NTDs. The MoH has also been upgrading HEWs' competencies to level IV (nurses and midwives) to manage such curative services. Furthermore, there have been changes in the infrastructure and commodity standards of HPs to meet service requirements (7,14,16, 26, 27). Despite all the aforementioned changes, rural communities still demanding adult and child curative services. The bypassing of the HPs may also have implications of mistrust in the capacity of HEWs to respond to the demands of rural communities towards quality curative health services (14).

HEPs' service delivery through home visits, HPs, and outreach was thought to be relevant. Service uptake through home visits was high in some studies. Other country-level studies also show that HP-based service utilization (9, 11, 14) (28, 29). Women advised by HEWs during pregnancy were highly likely to attend ANC and post-natal care (PNC) (29). HEWs' home visit practices improved early screening and detection of TB cases (30), utilization of HIV testing (31), and use of latrines (32). Access to ICCM, immunization, family planning services, and treatment of pneumonia had also been significantly improved in the HPs (6, 16). However, home visits and HP-based service delivery have been declining over time due to HEWs' workload and engagement in non-health activities (14, 27). Similarly, the community's demand for curative services increased despite the government's increase of HEWs' time to the HP from 25% to 50% (14, 27). It is revealed that the outreach is important in agrarian settings; but it is not working well for pastoralists due to high mobility.

Our study found that the current HEP packages are still relevant in addressing the rural community's priority health needs. HEP's service provision through home visits by HEWs, at HP level, and the outreach modality were all relevant. Although all the current HEP packages are relevant to rural communities' health needs and the government has made continuous adaptations in the packages, communities are still demanding advanced curative services in HPs. This may require rethinking of the current HEP standards against the universal primary healthcare coverage. There is also a need to reshape the relevant outreach service modality for pastoralist communities.

This study has a strength of responding to the overall question of HEP's relevance to the community's health needs for the first time. The assessment has also a limitation that it did not measure whether the HEP packages are implemented well or not.

## Figures and Tables

**Figure 1:**
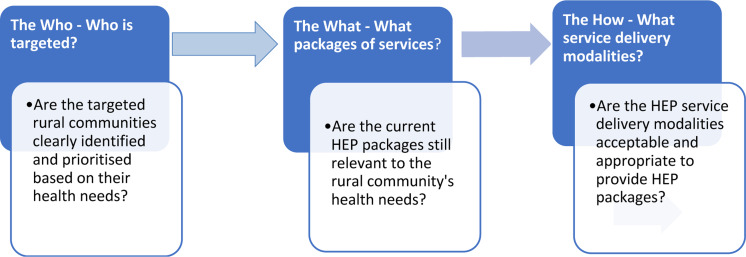
Framework for appropriateness or relevance of humanitarian programs (26)

## Data Availability

The datasets used for this study are available upon formal request from the Ministry of Health and MERQ Consulting PLC.
